# Humoral immunogenicity assessment after receiving three types of SARS-CoV-2 vaccine

**DOI:** 10.1038/s41598-023-47611-w

**Published:** 2023-11-18

**Authors:** Niloofar Najafi, Hoorieh Soleimanjahi, Lida Moghaddam-Banaem, Mohammad Reza Raoufy, Shadab Shahali, Anoshirvan Kazemnejad, Zeynab Nasiri

**Affiliations:** 1 Department of Virology, Faculty of Medical Sciences, Tarbiat Modares University, P.O. Box 14115-331, Tehran, Iran; 2 Department of Reproductive Health and Midwifery, Faculty of Medical Sciences, Tarbiat Modares University, Tehran, Iran; 3 Department of Physiology, Faculty of Medical Sciences, Tarbiat Modares University, Tehran, Iran; 4 Department of Biostatistics, Faculty of Medical Sciences, Tarbiat Modares University, Tehran, Iran

**Keywords:** Inactivated vaccines, Live attenuated vaccines, SARS-CoV-2

## Abstract

Several vaccines have been developed against SARS-CoV-2 and subsequently approved by national/international regulators. Detecting specific antibodies after vaccination enables us to evaluate the vaccine’s effectiveness. We conducted a prospective longitudinal study among members of Tarbiat Modares University of Tehran, Iran, from 4 September 2021 until 29 December 2021. We aimed to compare the humoral immunogenicity of 3 vaccine types. Participants consisted of 462 adults. Anti-SARS-CoV-2 receptor-binding domain [RBD] IgG titer was compared in 3 groups, each vaccinated by available vaccines in Iran at the time: Oxford/AstraZeneca, COVIran Barekat, and Sinopharm. The median IgG titer was: 91.2, 105.6, 224.0 BAU/ml for Sinopharm, COVIran Barekat and Oxford/AstraZeneca respectively after the first dose; 195.2, 192.0, 337.6 BAU/ml after the second one. We also analyzed the frequency of antibody presence in each vaccine group, in the same order the results were 59.0%, 62.6% and 89.4% after the first dose and 92.1%,89.5% and 98.9% after the second. The comparison of results demonstrated that AstraZeneca vaccine is a superior candidate vaccine for COVID-19 vaccination out of the three. Our data also demonstrated statistically significant higher antibody titer among recipients with an infection history.

## Introduction

The COVID-19 outbreak was first documented in December, 2019 in Wuhan, China. According to World Health Organization (WHO) report Sars-Cov-2 virus had already caused 771,549,718 confirmed cases of COVID-19 and 6,974,473 deaths, by October 25th 2023. As of October 21th 2023, a total of 13,533,465,652 vaccine doses were administered worldwide. meanwhile in Iran, morbidity and mortality rates were 7,619,981 and 146,480 respectively. A total of 155,445,801 vaccine doses were administered by 14 August 2023^1^.

During this time, safe and effective vaccines were added to WHO’s “Emergency Use List”, and multiple vaccines received authorization for emergency use by their national and/or international regulators^2^. Meanwhile, vaccine distribution in the developing countries were slow, and they were not close to optimal vaccination coverage; As a result, vast groups of unvaccinated people may play a critical role in COVID-19 circulation and lead to new variants evolution which can potentially be vaccine-resistant. So, swift worldwide mass vaccination with efficient vaccines is invaluable^3^. During SARS-COV2 pandemic, mutations in spike protein potentially increased transmission of the virus and its likelihood of escape from the antibodies raised against the parental sequence used in the administrated vaccines^4,5^. Although mutations can diminish the efficacy of antibodies produced against the original spike sequence, vaccines are recommended for public use in order to increase the global herd immunity^6^.

AstraZeneca vaccine was developed through the cooperation of Oxford University and AstraZeneca (ChAdOx1). It is based on genetic modifications to replication-defective adenoviruses that are inactivated due to deletion of the E1 gene, which is replaced with the spike gene of SARS-COV-2^7^. Spike protein is expressed on the surface of virus particle, triggering both antibody and T cell responses that can be protective against COVID-19. A safer alternative platform is utilizing inactivated vaccines. Sinopharm and COVIran Barekat vaccines were both produced based on the killed/inactivated virus strategy. The principal advantage of these vaccines is their similarity to the natural infection, which may induce a stronger and long-lasting immune response^8,9^. A meta-analysis containing 54 studies from 18 different countries, comprising about 12 million individuals, observed that 6–8 months after recovery the prevalence of SARS-CoV-2 specific immunological memory remained high. In fact, nearly 90% of recovered individuals had evidence of immunological memory of SARS-CoV-2 at 6–8 months after recovery^10^.

It is necessary to update the current vaccines to match the circulating SARS-CoV-2 variants. We believe that an imperative instrument to tracing infected individuals and saving their lives is the development of sensitive and specific detection methods for mass screening and testing as well as using effective vaccines^11,12^. At the time of this study, health-related challenges of COVID-19 include lack of access to vaccines in several countries and rejection of those who do have access to it^3^, the lack of an effective specific antiviral treatment^13^, and the possibility of transmission of the virus to pets and farm animals^14,15^.

Today, scientists and governments have realized the need for effective methods for diagnosing viruses such as SARS-CoV-2 at the early stages of their spread. Besides, to control the SARS-CoV-2 spread, it is important that vaccines with high efficacy and accessibility be available worldwide^16^.

Several factors may affect the protection offered by vaccines, including vaccine platforms type, SARS-CoV-2 variants, age and immunity status of the vaccine recipient, infection history, dosing intervals, vaccine storage and transportation capacity^17,18^.

Several serum-based evaluations were performed to reflect humoral immunity status which is often correlated with poor or strong responses obtained during infection or vaccine administration. The receptor binding domain (RBD) is responsible for binding to the ACE2 receptor on the cellular membrane, and the target protein for evaluating humoral and cellular immunity for vaccine efficacy evaluation^19,20^. Antibody testing for SARS-CoV-2 allows us to compare vaccine efficacy among participants with or without past exposure to the virus, even after it has been cleared by the immune system. A study indicated that individuals with pre-exposure to COVID-19 produced more antibodies against COVID-19 after vaccination. Furthermore, extensive exposure to the virus and prolonged duration of the illness increases the chance of producing antibodies against all strains of SARS- CoV-2^21^.

The exact effectiveness of vaccines against SARS-CoV-2 remains unknown. This study evaluates SARS-CoV-2 antibody surveillance in a normal academic population in Iran. It was designed to compare the efficacy of two international COVID-19 vaccines against one that is produced in Iran, by measuring levels of serum antibody produced by them. All of these vaccines were commonly used for mass vaccination in Iran. RBD domain of spike protein was used in ELISA assays to detect antibody titer of SARS-CoV-2.

In this study, distribution of age, sex, and history of infection of SARS-CoV-2 was compared in participants receiving the 3 vaccines types, and their potential effects on the produced antibody levels were assessed.

The efficacy of adenovector-based vaccines such as AstraZeneca and killed whole virion vaccines such as Sinopharm and COVIran Barekat was evaluated by serological assessment in individuals with or without prior infection by SARS-CoV-2. The humoral immunogenicity of these vaccines was compared by measuring the serum anti-SARS-CoV-2 anti-RBD antibodies levels.

## Results

### Demographic characteristics of participants

A total of 462 participants were included in this study, who received 3 different types of approved and available SARS-CoV-2 vaccines in Iran. The participant's ages ranged from 20 to 70 years old with a median of 37 (IQR 30–45), 44.2% of them were female, and 10.6% of all of the participants had priorSARS-CoV-2 infection history. In terms of age, sex, or infection history distribution, there were no statistically significant differences observed among the participants in the 3 vaccine groups. The demographic characteristics of participants are demonstrated in Table 1. Out of 462 participants, 301 returned for secondary examinations. Their demographic information is demonstrated in Table 2.

**Table 1:** Demographic characteristics of the participants receiving the first dose of COV2-vaccination (n = 462).

Qualitative variables		AstraZeneca		COVIran Barekat		Sinopharm		Total	
		Frequency	Percentage	Frequency	Percentage	Frequency	Percentage	Frequency	Percentage
Sex	Female	63	44.7	62	40.0	79	47.6	204	44.2
	Male	78	55.3	93	60.0	87	52.4	258	55.8
	Total	141	100	155	100	166	100	462	100
Infection history	Negative	120	85.1	144	92.9	149	89.8	413	89.4
	Positive	21	14.9	11	7.1	17	10.2	49	10.6
	Total	141	100	155	100	166	100	462	100
		Median (IQR 25–75)							
		AstraZeneca		COVIran Barekat		Sinopharm		Total	
Age (years)		33 (27–40)		40 (31–50)		38 (30–45)		37 (30–45)	

SD, standard deviation.

**Table 2:** Demographic characteristics of the participants receiving the second dose of COV2-vaccination (n = 301).

Qualitative variables		AstraZeneca		COVIran Barekat		Sinopharm		Total	
		Frequency	Percentage	Frequency	Percentage	Frequency	Percentage	Frequency	Percentage
Sex	Female	40	42.1	48	45.7	48	47.5	136	45.2
	Male	55	57.9	57	54.3	53	52.5	165	54.8
	Total	95	100	105	100	101	100	301	100
Infection history	Negative	82	86.3	97	92.4	91	90.1	270	89.7
	Positive	13	13.7	8	7.6	10	9.9	31	10.3
	Total	95	100	105	100	101	100	301	100
		Median (IQR 25–75)							
		AstraZeneca		COVIran Barekat		Sinopharm		Total	
Age (year)		33 (26–40)		41(31.5–52)		40 (32–48)		38(30–48)	

SD, standard deviation.

### Humoral immunogenicity assessment

Serum antibody titers were measured one month after each of the first and second vaccine inoculation. The median of antibody titers and IQR are demonstrated in Table 3. Because the non-normal distribution of antibody titers the non-parametric analysis, Kruskal–Wallis, was used to compare antibody titers among the 3 vaccine types, which showed P-values < 0.001 after both the first and the second dose of vaccination. Since this study includes three vaccine types, in order to do pairwise comparisons and determine the significant differences in their effectiveness, the Bonferroni Correction test was applied. This test showed that the difference in antibody titers in the COVIran Barekat-Sinopharm pair had an adjusted P-value of 1.000 which is not significant, while Sinopharm-AstraZeneca and COVIran Barekat-AstraZeneca pairs both had P-values < 0.001. As was expected, since Sinopharm and COVIran Barekat both use a similar production strategy, killed whole virion vaccine, induced similar antibody titers.

**Table 3:** Comparison of antibody titers after the first and second dose of COV2-vaccinations.

Vaccine type	Titer1^a^ (n = 462)	P-value	Titer2^b^ (n = 301)	P-value
	Median (IQR 25–75)		Median (IQR 25–75	
AstraZeneca	224.0 (68.8–396.8)	< 0.001	377.6 (275.2–416.0)	< 0.001
COVIran Barekat	105.6 (16.0–284.8)		192.0 (72.0–297.6)	
Sinopharm	91.2 (16.0–276.8)		195.2 (73.6–302.4)	

IQR, interquartile range; ^a^; antibody titer (BAU/ml) before the second dose of vaccination; ^b^; antibody titer (BAU/ml) 1 month after the second dose of vaccination. Significance values have been adjusted by the Bonferroni correction for multiple tests (Sinopharmm-COVIran Barekat 1.000, Sinopharm-AstraZeneca < 0.001, COVIran Barekat-AstraZeneca < 0.001) for both of measurements.

The frequency and percentage of participants who tested positive for anti-SARS-CoV-2 anti-S1-RBD antibody in each vaccine group are shown in Table 4. Chi-Square test was utilized to survey for any significant differences between the three different vaccine groups. The results demonstrated a statistically significant difference between positive results among participants in the 3 vaccine groups in both doses of vaccines, P-value < 0.001 for the first and P-value: 0.023 for the second dose were calculated.

**Table 4:** Comparison of antibody presence in different COV2-vaccines.

Vaccine type	Positive Result 1^a^ (n = 462)		P-value	Positive Result 2^b^ (n = 301)		P-value
	frequency	percentage	Chi-Square	frequency	Percentage	Chi-Square
AstraZeneca	126	89.4	< 0.001	94	98.9	0.023
COVIran Barekat	97	62.6		94	89.5	
Sinopharm	98	59.0		93	92.1	

^a^:Antibody presence in participants’ sera after the first dose of COV2-vaccination, ^b^: Antibody presence in participants’ sera one month after the second dose of COV2-vaccination.

### Impact of infection history, age and sex

We also assessed the impact of infection history on antibody titer through the Mann–Whitney U test for each vaccine group. After the first vaccination, distribution of antibody titer was significantly different based on infection history in AstraZeneca, Sinopharm, and the overall groups. Antibody titers were higher in participants with prior covid-19 infection. On the contrary, it was the same across categories of infection history for COVIran Barekat. After the second dose, compared to non-infected participants, our data demonstrated significant differences in antibody titers among recipients with an infection history, only in AstraZeneca and overall groups (Table 5). Spearman’s method was used to assess the correlation between antibody titer and age for each vaccine group. Besides, we evaluated the influence of sex on antibody titer by Mann Whitney U test for each vaccine group. Both analyses failed to demonstrate statistically significant relationships.

**Table 5:** The effect of infection history on antibody titer in 3 vaccine types.

Vaccine type	After the first dose			After second dose		
	Antibody titer (BAU/ml): Median (IQR 25–75)			Antibody titer (BAU/ml): Median (IQR 25–75)		
	With Inf.h	Without inf.h	P-value	With Inf.h	Without inf.h	P-value
AstraZeneca	796.8 (376.0–820.0)	152.0 (60.8–387.2)	< 0.001	419.2 (617.6–411.2)	366.4 (252.8–412.8)	< 0.001
COVIran Barekat	150.4 (51.2–323.2)	105.6 (16.0–278.4)	0.232	163.2 (64.8–381.6)	192.0 (72.0–297.6)	0.800
Sinopharm	275.2 (166.4–315.2)	57.6 (15.2–262.4)	0.003	264.0 (134.4–389.6)	176.0 (72.0–300.8)	0.159
Overall	320.0 (212.8–796.8)	105.6 (20.0–299.2)	< 0.001	384.0 (211.2–422.4)	235.2 (96.0–371.2)	0.002

Inf.h, infection history.

## Discussion

Humoral immunity is a major factor which plays a critical role in vaccines effectiveness. Despite the fact that the exact neutralizing response is investigated thorough cVNT, which requires biosafety level 3 equipment, Anti-SARS-CoV-2 anti RBD titer can be measured to evaluate humoral responses after infection or vaccination. The sVNT is practical in numerous studies on features of COVID-19, including vaccine efficacy during preclinical and clinical trials of different vaccine candidates and monitoring neutralizing antibody titers in vaccine recipients after mass vaccination^7,22,23^.

This was a prospective longitudinal study performed on 462 adult participants each receiving 2 doses of either AstraZeneca, COVIran Barekat or Sinopharm vaccines. One month after the injection offirst dose, a significant statistical difference was shown when comparing inactivated vaccine recipients (COVIran Barekat or Sinopharm) with participants who received AstraZeneca vaccine, both in the antibody presence (AstraZeneca 89.4%, COVIran Barekat 62.6%, and Sinopharm 59.0%, P-Value < 0.001) and the median antibody titer (AstraZeneca 224.0 (IQR 68.8–396.8) BAU/ml, COVIran Barekat 105.6 (IQR 16.0–284.8) BAU/ml, and Sinopharm 91.2 (IQR 16.0–276.8) BAU/ml, P-Value < 0.001). Also, the same results were obtained after the injection of the second dose, antibody presence were as follows: AstraZeneca 98.9%, COVIran Barekat 89.5%, and Sinopharm 92.1% (P-Value < 0.001). Also, the median antibody titer was significantly higher in AstraZeneca recipients (AstraZeneca 377.6 (IQR 275.2–416.0) BAU/ml, COVIran Barekat 192.0 (IQR 72.0–297.6) BAU/ml, and Sinopharm 195.2 (IQR73.6–302.4) BAU/ml, P-Value < 0.001). Inevitably, the protection offered by neutralizing antibodies through vaccination can be varied due to the differences in vaccine platforms and the interval of doses. Our findings demonstrated that Astra Zeneca has led to higher immunogenicity compared to other available vaccines in Iran.

An Iranian cohort monitoring study has revealed that when comparing AstraZeneca recipients with inactivated vaccines recipients, although the former has a significantly higher immunity during the interval of the first and second dose, after the second dosage no significant difference was found among different vaccine platforms. They concluded that SARS-CoV-2 vaccines, while unable to prevent the infection, can significantly reduce mortality rate^24^. These results are in line with our findings about the higher anti-SARS-CoV-2 anti RBD IgG detection rate and titer in AstraZeneca recipients.

It is obvious that antibody titers, post-infection or vaccination, cause a range of protective effects specifically by reducing the mortality rate. A study estimated that the required neutralizing antibody titer to completely prevent symptomatic viral infection is approximately sixfold higher compared to what is required to prevent severe outcomes of SARS-CoV-2 infection^25^.

In this study, we assessed antibodies in participants infected or not infected early in the pandemic during alfa and delta variants circulation time. The assessment of serum antibody activity after the first dose of SARS-CoV-2 vaccination (in September 2020) showed that participants who had previously confirmed infection in each vaccine group, or overall had a significantly higher amount of antibody titer as demonstrated in the RBD-based ELISA assay, except for COVIran Barekat group, which might be due to the low number of infected persons in this group: 11 out of 155 (7.09%). Moreover 2 of 11 COVIran Barekat recipients who had prior infection history, did not produce antibodies after the first vaccine administration. Considering that both of them were young (a 30 years old female and 38 years old male), they might be non-responders. The impact of these two samples was magnified in such a small fraction. Therefore, when we used Whitney U test for each vaccine group in order to investigate the impact of infection history, it led to a non-significant p-value for COVIran barekat group. These findings confirmed that additional antigenic exposure further improves antibody efficacy against SARS-CoV-2 variants. Protective antibodies from prior infection, with or without vaccination, induced antibodies against the spike protein to interfere with its function^26^. The antibody response is a crucial aspect of adaptive immunity against viral infections. Based on the predominant isotypes and profiles of somatic hypermutations of the antibodies, the humoral immune response can be divided into two phases. In the extrafollicular (EF) phase, B cells are activated and quickly differentiate into plasma cells in foci outside of the follicle within a few days after the infection. They produce antibodies that contain few somatic hypermutations but can still have reasonably high affinities and neutralize the virus^27^. Another study revealed the chances of reinfection are significantly lower in individuals who tested positive for antibodies. Furthermore, getting vaccinated after a SARS-CoV-2 infection enhances the impact of this response. Research has found that neutralizing antibodies are present in 99% of people infected with SARS-CoV-2 before. It is believed that the antibodies could provide strong protection against infection and reinfection in individuals without COVID-19 infection history, but were vaccinated previously^28^.

Many scientists are of the same concern about the waning of neutralizing antibody titers induced through prior infection or vaccination. Besides, the continuous emerge of new Variants Of Concern (VOC) of SARS-CoV-2 due to its broad spread and frequent mutation, has caused several neutralizing antibodies to lose their antiviral activities. However, neutralizing antibodies can recognize different epitopes in spike protein and some of them can neutralize new VOCs. As a result of that, pervious immunity induced by infection or vaccine might alleviate infection. It was suggested that, in order to decrease the VOC’s chance of escape from vaccine induced neutralizing antibodies, future vaccines must consider different groups of epitopes of RBD^29^.

The next step is to develop vaccines which are able to prevent infection completely. Mucosal immunity inducing vaccines might be effective in preventing infection and restricting viral transmission, however, there are still a number of obstacles to overcome to reach their full potential^30^. Mucosal vaccines have been successful in protecting against diseases, but there are still limitations and risks associated with their clinical development. One concern is that mucosal surfaces are frequently exposed to harmless foreign substances and commensals, which can lead to tolerogenic immune programs that may result in weak induction of pro-inflammatory responses. Effective adjuvants can help break tolerance, however, there are limited mucosal adjuvants that are safe for human use and many require further testing to demonstrate their safety and efficacy. Additionally, antigen dilution can hinder vaccine delivery at mucosal surfaces, where ciliated cells quickly clear away debris and pathogens in a protective mucous layer^31^.

In this study, previous infection history was significantly related to higher anti SARS-CoV-2 anti RBD IgG titers (P-Value < 0.001 after the first dose, P-Value: 0.002 after the second dose). Many studies subscribe to this theory, which states infection history can boost vaccine effectiveness and lead to an increase in antibody titer and furthermore can shorten the interval of vaccine injection and rises in antibody titer^21,32,33^.

After categorizing participants based on their sex or age, no significant difference was observed in the median antibody titer. AstraZeneca clinical trial phase 2/3 data has not shown any significant difference in neutralizing antibody titer among different age groups, after the second dose injection. The data also demonstrated that neutralizing antibody has been detected among 99% of participants 14 days after the second dose injection^34^. However, another meta-analysis including approximately 7 million participants from 18 studies, has found that vaccine effectiveness in prevention of severe SARS-CoV-2 infection was significantly lower among participants older than 65 years old^35^. It might be due to the fact that different vaccine types were investigated in that study; since it investigated mRNA-based vaccines and Johnson and Johnson vaccine. Moreover, our study’s aim was to evaluate antibody titer among university staff and students, leading to 84% of our participants being 50 years old or younger, which may result in limitations.

The limitations of this study are: it did not consider children and elderly citizens (> 70), cell-mediated immune responses were not assessed, and last but not least, 34% of the participants in the first sampling did not return to partake in the second round of it.

Although, the emergence of new VOCs and waning neutralizing activity of vaccines has faced researchers with a dilemma, how often should we administer SARS-CoV-2 vaccines and more importantly how safe are these repeated injections? Further studies need to address the problems of safety and efficacy of the developed and the under-development vaccines against SARS-CoV-2, which are achievable through real-world surveys.

## Conclusion

This study demonstrated that AstraZeneca vaccine induced higher humoral immunogenicity when compared to the inactivated vaccines (COVIran Barekat, Sinopharm), since it had more positive cases of antibody presence and higher antibody. Also, results of this study, agreeing with several other studies, indicated that infection-induced immunity combined with vaccination results in higher levels of protection against COVID-19 which may prevent severe illness, hospitalization and mortality. Therefore, our findings suggested that vaccination may be more crucial for people who haven’t been infected by SARS-CoV2.

## Methods

### Study design

This research was a prospective longitudinal study, performed among 462 staff and students of Tarbiat Modares University, Tehran, Iran. It was conducted from 4 September 2021 until 29 December 2021. The **Delta** variant of SARS-CoV-2 was the predominant strain during this period.

All university staff and students who attended the health center of Tarbiat Modares University to get their second dose of vaccination with Oxford/AstraZeneca, COVIran Barekat or Sinopharm, and were willing to participate in the study were enrolled. 2 ml of peripheral venous blood sample was collected for serological survey. The participants were recalled 1 month after the second injection for the second round of blood sample collection.

### Ethical considerations

This study was performed in accordance with the Declaration of Helsinki and was approved by the Ethics Committee of Tarbiat Modares University of Tehran (code number: IR.MODARES.REC.1400.138).

The researchers explained the method and aim of the study. The participants signed the formal informed consent form and were able to withdraw from the study at any time. They were also able to communicate with the research team for more information about the tests and results, or ask any questions related to this study.

### Data collection

At first, demographic data of participants was gathered via filling out an online questionnaire (including age, sex, education, employment status, and having a history of asymptomatic or symptomatic Covid morbidity, covid symptoms in close co-habitants). The participants were put through an online survey one-week after each round of vaccination to self-report any experienced side effects.

### Sample collection

For each participant, peripheral venous blood samples (2 ml) were collected and transferred to the virology laboratory of Tarbiat Modares University of Tehran. Sera were separated from whole blood by centrifuging samples for 10 min and were stored at − 20 ℃ prior to detecting SARS-CoV-2 antibody level.

### Serological assessment by ELISA Kit

Since our aim was to survey the antibody response in vaccinated individuals, Enzyme-Linked Immunosorbent Assay (ELISA) was used for serological assessment, which detects Anti-SARS-CoV-2 Spike Protein RBD Antibody. In this research Chemobind Anti-SARS-CoV-2 Spike Protein RBD Antibody detection kit was used. We used RBD-based kits representing frequently used antigens for their reactivity to human IgG antibodies using ELISA assay in serum from COVID-19 vaccinated individuals. Besides, suspected results of the ELISA kit (according to the manufacturer’s instructions, samples containing Antibody titer from 25.6 to 35.2 BAU/ml are considered as suspect and must be double-checked) were checked again they were also compared against the EUROIMMUN IgG ELISA test. The homemade kit (Chemobind developed by a knowledge-based enterprise supervised by Tehran University of Medical Sciences. It has been used in other studies and its results has been compared with well-known kits such as Euroimmun^36^) had 6 calibrators with different dilutions (1, 10, 20, 40, 80, 120), each equal to a particular dilution of antibody in sera. The kit included negative and positive controls. The negative and positive controls included inactivated human serum diluted via diluting buffer, devoid of Anti-SARS-CoV-2 RBD Antibody for the negative and containing it for the positive control.

The serum samples were thawed, vortexed, and diluted before testing. In each run, every calibrator along with the positive and the negative controls was used duplicated. The kit was used in accordance with the manufacturer's protocol. Based on the optical density of calibrators and their dilutions a standard curve was plotted in each run. Anti-SARS-CoV-2 RBD antibody titer was calculated for each participant in accordance with the OD measurements for samples and the plotted standard curve of its run using the Curve Expert application. Samples containing higher antibody titer than the most concentrated calibrator > 120 were diluted and tested again.

### Statistical analysis

Data entry was done using Microsoft Excel and statistical analysis was performed through IBM SPSS version 28. The collected data were analyzed using Kruskal–Wallis test with Bonferroni correction, Chi-square test, Mann Whitney U test, and Pearson correlation analysis test.

## Data Availability

The corresponding author will provide supporting data through email in case of any editor or referee requests it.
